# Connectivity mapping using a combined gene signature from multiple colorectal cancer datasets identified candidate drugs including existing chemotherapies

**DOI:** 10.1186/1752-0509-9-S5-S4

**Published:** 2015-09-01

**Authors:** Qing Wen, Paul O'Reilly, Philip D Dunne, Mark Lawler, Sandra Van Schaeybroeck, Manuel Salto-Tellez, Peter Hamilton, Shu-Dong Zhang

**Affiliations:** 1 Centre for Cancer Research and Cell Biology (CCRCB), Queen's University Belfast, Belfast, UK

**Keywords:** connectivity mapping, gene signature, systems approach, multiple datasets, colorectal cancer, irinotecan, etoposide

## Abstract

**Background:**

While the discovery of new drugs is a complex, lengthy and costly process, identifying new uses for existing drugs is a cost-effective approach to therapeutic discovery. Connectivity mapping integrates gene expression profiling with advanced algorithms to connect genes, diseases and small molecule compounds and has been applied in a large number of studies to identify potential drugs, particularly to facilitate drug repurposing. Colorectal cancer (CRC) is a commonly diagnosed cancer with high mortality rates, presenting a worldwide health problem. With the advancement of high throughput omics technologies, a number of large scale gene expression profiling studies have been conducted on CRCs, providing multiple datasets in gene expression data repositories. In this work, we systematically apply gene expression connectivity mapping to multiple CRC datasets to identify candidate therapeutics to this disease.

**Results:**

We developed a robust method to compile a combined gene signature for colorectal cancer across multiple datasets. Connectivity mapping analysis with this signature of 148 genes identified 10 candidate compounds, including irinotecan and etoposide, which are chemotherapy drugs currently used to treat CRCs. These results indicate that we have discovered high quality connections between the CRC disease state and the candidate compounds, and that the gene signature we created may be used as a potential therapeutic target in treating the disease. The method we proposed is highly effective in generating quality gene signature through multiple datasets; the publication of the combined CRC gene signature and the list of candidate compounds from this work will benefit both cancer and systems biology research communities for further development and investigations.

## Introduction

Drug discovery is a complex, lengthy and costly procedure. The entire process of bringing a new drug to market takes approximately 15 years, the average cost of which is estimated to be $1.8 billion [1]. There is an urgent need to develop drugs in a cost efficient way for the benefit of patients and drug developers [2]. One approach to this problem is to identify new uses for existing drugs, known as drug repurposing or repositioning, which can save production cost and time. As existing drugs are normally approved for human use, the identified new use can directly enter phase II clinical trials, saving at least 2 years of time and 40% of the overall cost of the complete procedure [3]. DNA microarray technology enables high-throughput gene expression profiling with a large number of genes assayed simultaneously, which can be used as a powerful tool in drug discovery and clinical medicine [4]. The integration of microarray technology and innovative bioinformatics frameworks, such as connectivity mapping, is particularly useful in facilitating drug repositioning.

Connectivity mapping uses gene expression profiling to connect genes, diseases and small module compounds. It is a process whereby gene expression profiles of cell-line response to drugs are compared to query gene signatures, in order to evaluate similarity and identify potential drugs able to alter the gene signature's expression [5]. A functioning connectivity mapping framework includes three major constituents: a reference database, query gene signatures, and a matching algorithm. A **reference database **contains a collection of chemical-induced gene expression profiles, describing chemical-induced reference states. The reference database of the Broad Institute Connectivity Map 02 contains 6100 individual reference profiles for 1309 small molecule compounds, many of which were FDA approved drugs [6]. A **query gene signature **is a list of genes selected from biological experiments investigating a particular biological condition. Differentially expressed genes between disease and normal conditions are often used to form a signature for the disease. Connectivity mapping employs non-parametric pattern **matching algorithms **to compare the gene signature and the reference profiles. As a result of the comparisons, all drug profiles in the reference database will be given a connectivity score range from 1 to -1 representing their connections to the query signature. A drug with the closest profile in the same regulation direction will be the best candidate to enhance the biological condition of the query signature. Conversely, a drug with the closest profile in the opposite regulation direction may be an inhibitor to that condition.

Connectivity mapping was first developed by researchers at the Broad Institute in 2006. Since then, connectivity mapping has been employed in numerous studies, gaining wide recognition in drug discovery and development [7]. It has been applied in a large number of microarray and also next generation sequencing (RNA-Seq) studies for candidate therapeutics identification [7,8]. As an alternative framework of connectivity mapping, statistically significant connections' map (sscMap) was developed by Zhang and Gant in 2008, which adopted the concept and the reference profile data of the original CMap. In addition, sscMap includes a refined method in the mapping process by calculating p-values at the level of individual reference profiles, which consequently helps to control false positive findings. SscMap has been shown to be an effective and robust framework to achieve high accuracy in identifying potential drugs [9,10]. A GPU-enabled tool cudaMap [11], which implements a parallel computing model of the sscMap algorithms, can greatly reduce the processing time of connectivity mapping.

Connectivity mapping represents a valuable bioinformatic technique to identify potential drugs and to generate new biological hypotheses. Query gene signatures are created by end users as input to connectivity mapping analysis, so signature quality is critically important to the success of the process. However, so far relatively little research has been done on methods for creating high standard gene signatures for connectivity mapping, in particular when they are to be generated from multiple datasets. The limited guidance from the original developers of Connectivity Map suggested that they found gene signatures with between 10 and 500 genes performed well. Users of connectivity mapping normally use their own experience and knowledge to create their own query gene signature.

The general way to compile a gene signature starts with comparison of gene expression between normal and disease tissues, resulting in a list of differently expressed genes (DEGs) as a representation of the disease phenotype. However, the lists of DEGs from various gene expression profiling studies tend to be inconsistent. For the same biological condition, the gene signatures from different datasets can be very different. Some genes may be significant in one dataset but not in the others; the same genes may have different significance levels or even different regulation directions in different datasets. Taking intersection of the lists from independent studies is a common approach to this problem [12], but this overlapping process can often filter out important genes. For example, if a gene is significant across all datasets except one, the overlapping method will exclude this gene. Furthermore, as the number of datasets increases, it may lead to an empty overlapping list because no single gene is significant across all datasets. As more and more data become publicly available, how to take advantage of these data and wisely use them will become more challenging. It will be an important research direction in gene expression profiling to combine gene signatures from diverse research with similar settings to derive a robust and stable representation of the biological conditions of interest.

In this paper, we propose a useful method using signed and normalized ranks to combine results from multiple colorectal cancer datasets. Colorectal cancer is a major disease prevailing worldwide with high mortality. According to 2013 U.S. cancer statistics, the estimated number of new cases and deaths annually for colorectal cancer in the United States are 142,820 and 50,830 respectively. Both the diagnosis and death toll from this disease are among the top three of all cancers[13]. We focus on colorectal cancer with the intention to compile high-quality gene expression signatures to represent the disease, and subsequently apply connectivity mapping to find potential drugs for the treatment of the disease.

## Methods

### Datasets: Samples and genes selection

Datasets GSE21510, GSE41258 and GSE49355 were obtained from the Gene Expression Omnibus(GEO), a public gene expression data repository. These datasets contain both tumour and normal, paired or unpaired, stage I-IV CRC samples, with raw microarray expression data available. We selected only stage II, III, and IV and excluded stage I samples in our study.

Dataset GSE21510 includes in total 148 samples from 104 colorectal cancer patients who had surgical therapy between 2002 to 2007 at Tokyo Medical and Dental University Hospital, Japan [14]. 46 tumour and paired normal samples from 23 patients are available. Excluding 8 (4 pairs) stage I samples, we selected 38 (19 pairs) samples from this dataset: 16 (8 pairs) stage II, 14 (7 pairs) stage III, and 8 (4 pairs) stage IV samples.

Dataset GSE41258 was from the study of Sheffer et al [15] on patients with colonic neoplasia presented at Memorial Sloan-Kettering Cancer Center, New York between 1992 and 2004. Biological specimens used in the original study included primary colon adenocarcinomas, adenomas and corresponding normal mucosae from patients at a variety of clinical stages. There are 88 paired samples in this dataset, from which we selected 74 paired, stage II-IV samples for our analysis. The selected include 16 stage II, 22 stage III and 36 stage IV samples.

Dataset GSE49355 was from a prospective study at the Institut du Cancer de Montpellier, France during January 2000 and June 2004, which involved 50 colorectal cancer patients. Samples of normal colon, primary colon cancer and hepatic metastasis were collected during surgery and before chemotherapy [16]. We selected 30 paired primary tumour and normal samples from this dataset, all samples were at stage IV.

The platforms used for these datasets were Affymetrix HG-U133A (for GSE41258 and GSE49355) and HG-U133 Plus 2 (for GSE21510), measuring the expression of 22283 and 54675 probe-sets respectively. Between these two platforms, there were 22277 common probesets, and these became the selected genes analyzed in our study.

### Data processing and analysis

Raw data and series matrix files of GSE21510, GSE41258 and GSE49355 were downloaded from GEO and extracted with 7zip. Data were normalised and summarised using Bioconductor affy package MAS5 algorithm. Series matrix files were used to extract the clinical information such as phenotypes (normal or tumour) and stages. Samples were further clustered in each dataset according to their stages (2-3 or 4), stage 2 and 3 samples were grouped together as a combined group of 2*3 and stage 4 samples formed a separate group. As a result, five sub-datasets were created: GSE21510S2*3 (n = 30), GSE41258S2*3 (n = 38), GSE21510S4 (n = 8), GSE41258S4 (n = 36) and GSE49355S4 (n = 30). Within each sub-dataset, half of the samples were primary tumours and the other half were the corresponding paired normals. Table 1 summarizes the sample and platform information for each dataset.

**Table 1:** Information of datasets used.

Dataset	Stage	Samples	Platform	TotalGenes	SignificantGenes
GSE21510	2-3	30(15pairs)	HG-U133Plus2	54675	4025
GSE41258	2-3	38(19pairs)	HG-U133A	22283	929
GSE21510	4	8(4pairs)	HG-U133Plus2	54675	7
GSE41258	4	36(18pairs)	HG-U133A	22283	663
GSE49355	4	30(15pairs)	HG-U133A	22283	1323
Combined Dataset	2-4	142(71pairs)		22277	4757

### Ranking method

To identify differentially expressed genes between the two biological conditions, normal versus tumour, we carried out paired-sample T-test on each gene in these datasets individually. As a result, each gene had a p-value from the T-test in each dataset. A stringent threshold p-value was set as 1*/π*_0_*N *, where *N *was the total number of genes analysed (N = 22277), *π*0 the proportion of non-differentially expressed genes, which could be accurately estimated using the Zhang-Gant method [17,18]. Setting such a stringent threshold was to control the expected number of false positives as 1 in multiple testing. All genes in each dataset were then divided into 2 groups: statistically significant group and non-significant group. Genes with a p-value smaller than the threshold were put in the significant group; the rest in the non-significant group.

We developed the following method to combine scores from different datasets and select significant genes. The ranking and scoring of all genes in an individual dataset is described in steps 1-5 below:

1. All genes in the statistically non-significant group will have 0 score.

2. Genes in the statistically significant group will have a score from *M *to 1 according to their significance order, the score will be normalised by *M *, where *M *is the number of significant genes identified in each dataset [see note (a) below].

3. The score for the *i*th gene in the ordered list is calculated using the formula:

Score = (M-i+1)/M

4. Apply a biological significance threshold and set their score to 0 for those genes with a fold change less than 2 in the statistically significant group.

5. Assign a sign to each score, giving "+" if the gene is up-regulated or "-" if down-regulated [see note (b)].

6. Sum up the scores of each gene across all datasets, so that each gene has a total score.

7. Sort all genes according to the absolute value of their total score in descending order, the gene with the highest absolute total score (the most significant gene) will be on the top.

8. Finally use the gene signature progression procedure (see the following section) with sscMap to decide the signature length.

Note:

(a) For different datasets, the number of statistically significant genes *M *is generally not the same. Normalising the scores by *M *makes them comparable across different datasets.

(b) Each score contains a sign, so if a gene regulates differently in different datasets, its per-dataset scores will counteract each other and its overall score is reduced.

The purpose of this ranking procedure is to identify genuinely significant genes across all of the datasets. Using this method, an overall top-ranked gene across multiple datasets must consistently have high ranking scores from these datasets, and must also have the same regulation direction in the majority of datasets. Only such genes are guaranteed to retain high overall scores.

Table 2 lists the top ranked gene in each individual dataset (FXR1, CDH3, COL11A1, HSPH1 and SMPD1), along with their scores in the other datasets. As can be seen from Table 2, depending on their individual scores across all datasets their overall scores are varied. CDH3 is the top gene in dataset GSE41258S2*3, but due to its less prominent positions in other datasets, the overall rank for this gene across five datasets is 25. FXR1 is the top gene in GSE21510S2*3 dataset, and because it is not significant in all other datasets, its overall rank is dropped to 1000. In fact, none of these five genes are in the list of top 20 genes by overall ranking. Table 3 shows the top 10 genes by overall ranking. A common feature of these top 10 genes is that each gene is consistently significant (and with the same regulation direction) across all datasets except GSE21510S4, which only returned 7 significant genes due to small sample size and low statistical power.

**Table 2:** Top 1 gene of each dataset and its scores in all other datasets.

ProbeID	GeneSymbol	GSE21510S2*3	GSE41258S2*3	GSE21510S4	GSE41258S4	GSE49355S4	TotalScore	OverallRank
201637 s at	FXR1	1	0	0	0	0	1	1000
203256 at	CDH3	0.6174	1	0	0.9400	0.9983	3.5557	25
37892 at	COL11A1	0.9990	0.8303	0	1	0.4707	3.3000	64
206976 s at	HSPH1	0.3651	0.9524	0	0	1	2.3175	262
216230 × at	SMPD1	-0.6184	-0.8424	-1	0	0	-2.4608	222

**Table 3:** Top 10 genes of the combined signature and their scores in all datasets.

ProbeID	GeneSymbol	GSE21510S2*3	GSE41258S2*3	GSE21510S4	GSE41258S4	GSE49355S4	TotalScore	OverallRank
203908 at	SLC4A4	-0.9933	-0.9847	0	-0.9895	-0.9883	-3.9557	1
207502 at	GUCA2B	-0.9904	-0.9973	0	-0.9797	-0.9826	-3.9500	2
207003 at	GUCA2A	-0.9627	-0.9989	0	-0.9822	-0.9513	-3.8951	3
205480 s at	UGP2	-0.9912	-0.9732	0	-0.9197	-0.9996	-3.8836	4
205950 s at	CA1	-0.9974	-0.8872	0	-0.9959	-0.9352	-3.8159	5
212942 s at	KIAA1199	0.9472	0.9962	0	0.8775	0.9926	3.8135	6
203961 at	NEBL	0.9788	0.9639	0	0.8508	0.9887	3.7821	7
219909 at	MMP28	-0.9332	-0.9995	0	-0.8856	-0.9613	-3.7796	8
213766 × at	GNA11	-0.9973	-0.9037	0	-0.9059	-0.9609	-3.7677	9
202370 s at	CBFB	0.9886	0.8933	0	0.9335	0.9522	3.7676	10

### Gene signature progression procedure

In the construction of a gene signature for connectivity mapping, there are often a large number of genes that passed the threshold p-value and were selected as significant genes. But including all the significant genes in a query signature might not be the best choice to describe the biological condition under study, since this may dilute the critical features of a biological condition. As the purpose of a gene signature is to capture the most important/prominent features of a biological state, we define the optimal signature length as the smallest number of genes from the top of the ranked list to achieve a target number of significant connections. To effectively utilize connectivity mapping, the drug hits in sscMap are used to guide the choice of gene signature length. We implement the following procedure to decide the length of a query signature.

1 First, as in the sscMap framework, we set $E_{f_{p}}=\text{1}$ as the expected number of false positives to tolerate among the drug hits. The threshold p-value for declaring significant connection is then set as $E_{{}_{f_{p}}}/N_{c}=\text{1}/\text{13}0\text{9}$, where *Nc *= 1309 is the number of small molecule compounds in the connectivity mapping database.

2 Set the target false discovery rate (FDR) as 0.10 (one in ten risk of false drug hit). The empirical FDR is calculated by $E_{f_{p}}/Ns$, where *N*_s_ is the number of significant drug hits.

3 Start from *m *= 1, where *m *denotes the number of genes included in a query gene signature.

4 Select the top *m *genes from the ranked list obtained from the differential expression analysis with multiple datasets discussed above.

5 Use the gene signature of the top *m *genes to query sscMap, and check how many drugs have significant connections to this query signature. As we are looking for potential drugs to inhibit the disease, we only consider compounds in the desirable direction of action, ie, those with negative connection scores.

6 If $\text{FDR}=E_{f_{p}}/N_{s}>0.\text{1}0$, increase the number of genes *m *by 1 and go to step 5.

7 Until FDR becomes *≤ *0.10, or the ranked significant genes from differential expression analysis have been exhausted, the process is stopped. At this point, the optimal length of the gene signature and at least 10 top significant drugs will be obtained as the result of gene signature progression.

## Results

### Significant genes result

Paired-sample T-tests were carried out on 5 subdatasets individually. The genes in each dataset were ranked and scored using the methods described. In dataset GSE21510S2*3, 4025 genes were identified as significant with a non-zero score. GSE41258S2*3 had 929 significant genes, GSE21510S4 had 7, GSE41258S4 had 663, and GSE49355S4 had 1323. The combined signature had 4757 significant genes with a non-zero score (Table 1). As a result of the ranking and progression procedures described above, the signature with the top 148 genes from the overall ranked list identified 10 significant drugs with a FDR *≤ *0.10, which was the pre-set FDR threshold. The supplementary information includes the full list of genes, their scores in individual datasets and their overall scores.

SLC4A4, GUCA2B, KIAA1199 and MMP28 are among the top 10 significant genes in the combined signature. Their differential expression in colorectal cancer have also been reported in the literature. SLC4A4 is the top gene in our combined signature, with the highest absolute overall score, but the sign of the score is negative, which is in agreement with data from the Cancer Genome Atlas (TCGA) showing that SLC4A4 mRNA level is decreased in colon adenocarcinomas [19]. GUCA2B, a gene coding for uroguanylin, has been found to be down regulated by 8-fold in adenoma [20]. It has also been proposed as a noninvasive biomarker for early CRC detection [21]. Moreover, radio-labeled uroguanylin analogs have been used in vivo to detect CRC [22]. KIAA1199 is the sixth significant gene in the combined signature, which is significantly up-regulated in the colorectal cancer tumour sample. It has been found that suppression of KIAA1199 weakens Wnt-signalling and inhibits the proliferation of colon cancer cells [23]. MMP28 (Matrix metalloproteinase 28) is the 8th significant gene in the combined signature with a negative score as it is significantly down-regulated in the colorectal cancer tumour samples. Consistent with our finding here, the downregulation of MMP28 in colorectal cancers has been validated in detailed analysis of MMP gene expression patterns [24]. All these relevant findings suggest that the 148 genes selected from progression procedure play important roles in colorectal cancer, therefore may be considered as potential therapeutic targets for drug development.

### Significant drugs result

**Figure 1:**
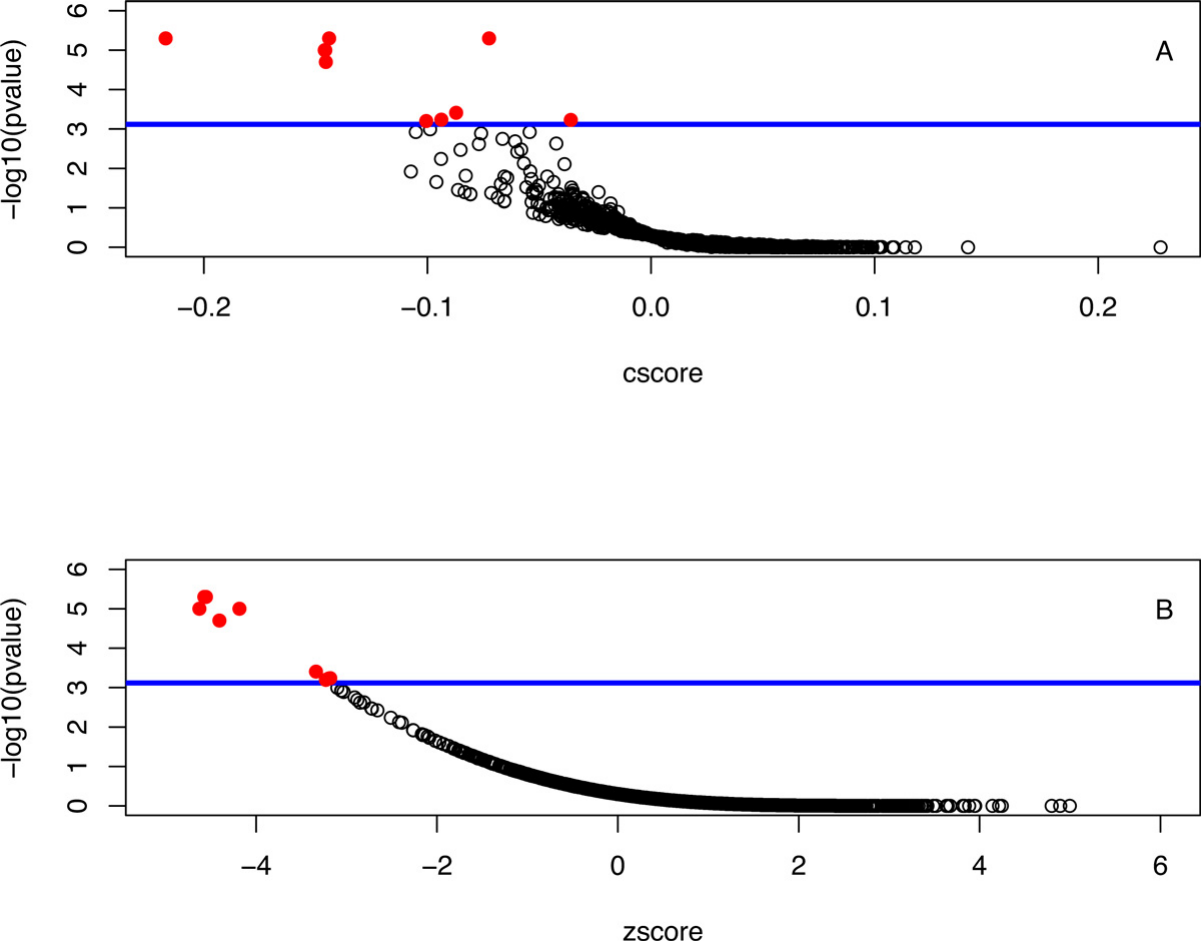
**Results of sscMapping using the 148-gene signature determined from the gene signature progression procedure**. Each data point represents a compound, with the raw connection score (cscore) or the normalized score (zscore) shown in (A) and (B) respectively. The blue line in the plot corresponds to the position of threshold p-value. Any data points above the blue line are drugs that have significant connections (solid red circles) to CRC gene signature.

The top 148 genes by the combined ranking formed the the optimal gene signature as determined by the gene signature progression method. This gene signature represented an accurate characterization of colon cancer disease phenotype, which returned from connectivity mapping 10 potential drugs for CRC treatment. These include trichostatin A, vorinostat, HC toxin, sodium phenylbutyrate, mycophenolic acid, irinotecan, etoposide, valproic acid, arachidonic acid, and rifabutin. Figure 1 shows the results of sscMapping using this CRC gene signature, where the significant drug hits are shown as solid red circles above the threshold blue line.

To test the robustness of the connections established between the query signature and the drug reference profiles, perturbation method was used to gauge the connection stability in connectivity mapping [25]. Gene signature perturbation analysis was performed with the 148-gene signature; perturbation stability scores were calculated for each drug. Table 4 shows the ten significant drugs identified and their perturbation stability scores. As shown in this table, six out of ten identified potential drugs received highest stability score 1. These include trichostatin A, vorinostat, HC toxin, sodium phenylbutyrate, mycophenolic acid, and irinotecan. The other four compounds (etoposide, valproic acid, arachidonic acid, and rifabutin) received perturbation stability score less than 1, with a minimum of 74%.

**Table 4:** The connections and perturbation stabilities of the 10 significant drugs obtained for the 148-gene signature.

Compound	Replicate	cscore	pvalue	zscore	PerturbStability
trichostatin A	182	-0.144	5.0E-06	-5.87	1.00
vorinostat	12	-0.146	1.0E-05	-4.63	1.00
HC toxin	1	-0.217	5.0E-06	-4.57	1.00
sodium phenylbutyrate	7	-0.072	5.0E-06	-4.56	1.00
mycophenolic acid	3	-0.145	2.0E-05	-4.41	1.00
irinotecan	3	-0.146	1.0E-05	-4.18	1.00
etoposide	4	-0.087	3.9E-04	-3.34	0.90
valproic acid	57	-0.036	5.9E-04	-3.19	0.78
arachidonic acid	3	-0.094	5.8E-04	-3.18	0.77
rifabutin	3	-0.100	6.3E-04	-3.23	0.74

**Figure 2:**
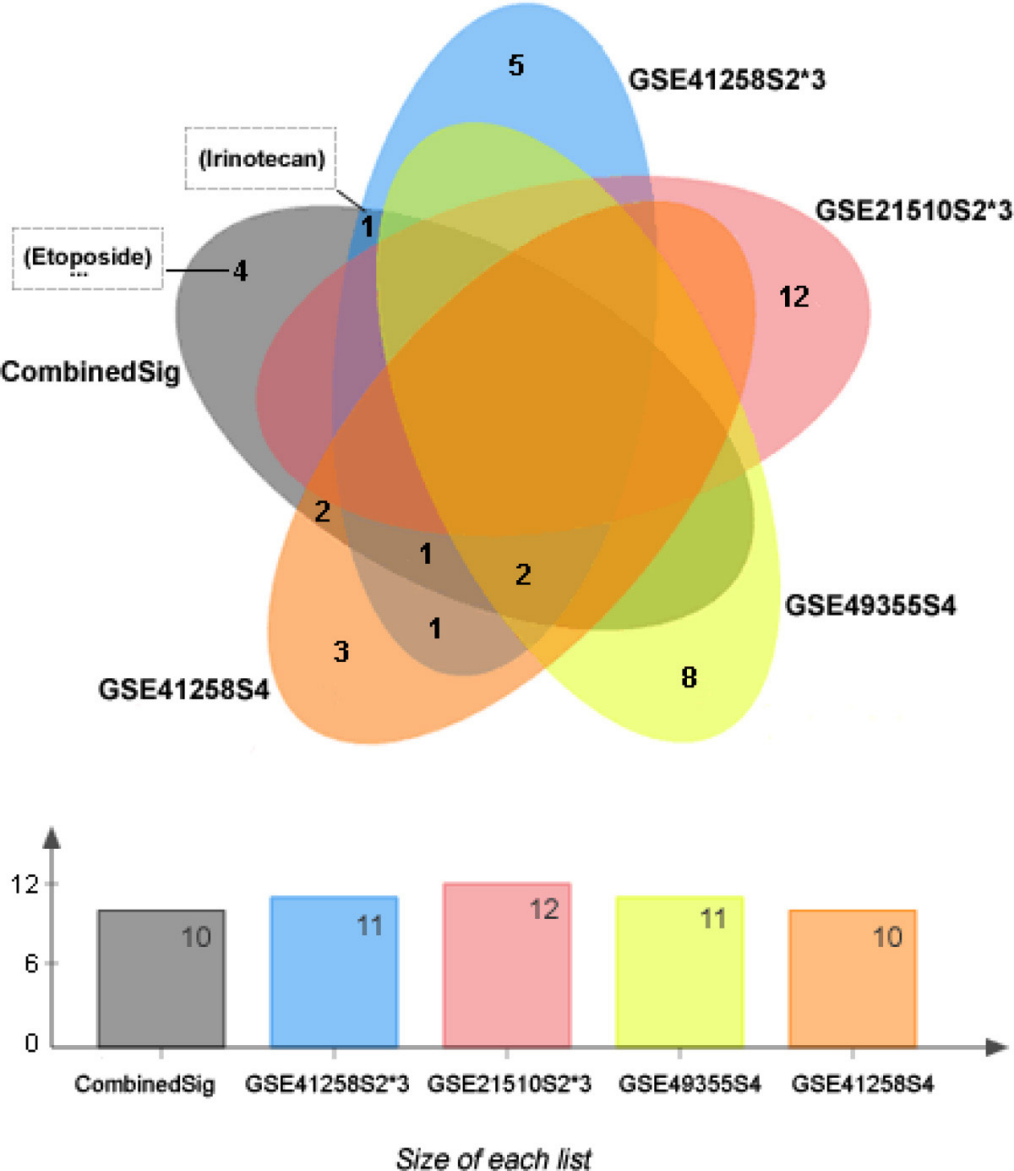
**The numbers of drug hits returned using gene signatures from individual datasets and from the combined 148-gene signature**. Note that only the combined gene signature drug list contains both irinotecan and etoposide, which are two existing CRC therapeutic drugs.

Among these 10 candidate compounds identified as potential drugs for colorectal cancer, irinotecan and etoposide are currently used to treat colorectal cancer already, pointing to the direction that other candidate compounds on this list are likely to have similar activity or power to treat the disease. To demonstrate the added values in combining multiple datasets, we also carried out connectivity mapping analysis on each dataset individually. The drug results from the combined signature are compared with that from each individual signature in a Venn diagram in Figure 2, which was created using jVenn [26]. The itemized drug results are listed in Table 5. As can be seen, although irinotecan also appeared in the results of dataset GSE41258S2*3, only the combined signature has both irinotecan and etoposide on its result list. Therefore, the drug results from the combined signature look much stronger than that from any individual signatures, suggesting that the combined signature provides a higher fidelity representation of the colorectal cancer phenotype.

**Table 5:** Drug results from combined and individual datasets.

CombinedSig	GSE41258S2*3	GSE21510S2*3	GSE49355S4	GSE41258S4
trichostatin A	irinotecan	letrozole	methylergometrine	trichostatin A
vorinostat	sirolimus	nystatin	vorinostat	HC toxin
HC toxin	trichostatin A	quipazine	0316684-0000	vorinostat
sodium phenylbutyrate	scriptaid	josamycin	nocodazole	rifabutin
mycophenolic acid	vorinostat	benzocaine	scriptaid	sodium phenylbutyrate
irinotecan	camptothecin	isocorydine	simvastatin	scriptaid
etoposide	dexamethasone	metampicillin	morantel	monorden
valproic acid	LY-294002	ethisterone	edrophonium chloride	alvespimycin
arachidonic acid	fludroxycortide	fluticasone	trichostatin A	dexamethasone
rifabutin	methylbenzethonium chloride	hycanthone	trazodone	tanespimycin
	sodium phenylbutyrate	tenoxicam	butein	
		adenosine phosphate		

Notice that in Table 5, there is no drug list for GSE21510S4, because the gene signature from this individual dataset did not return any significant drug hits in our connectivity mapping analysis. Due to the small sample size of this sub-dataset, with only 8 (4 pairs) samples, only 7 genes were identified as DEGs with a non-zero score. Consequently the contribution of this sub-dataset to the final combined signature was rather limited, as demonstrated in Table 3, where GSE21510S4 had no effects. This is actually a desirable characteristic of the multi-dataset method proposed in this paper, because low-powered or under-powered datasets will automatically have less influence on the final combined results.

## Discussions

Using multiple CRC datasets, combined with meta-analysis and connectivity mapping, this work was aimed at finding the connections between a molecular signature of colorectal cancer and possible drug treatments. Among the 10 significant drug hits in our list, irinotecan and etoposide are chemotherapy drugs currently being used to treat colon cancer. This illustrates the value of connectivity mapping to extract these complex relationships from public datasets. This would suggest that the molecular signature of colorectal cancer presented here could be a more precise representation of the biological phenotype. The robust connections between colorectal cancer and potential drugs obtained through connectivity mapping could be strong candidates for further investigation.

It is interesting to note that among the 10 candidate drugs, five are histone deacetylase inhibitors: trichostatin A, vorinostat, HC toxin, sodium phenylbutyrate, and valproic acid. Histone deacetylases (HDACs) are enzymes having critical roles in the regulation of gene expression, and they have emerged as promising targets in cancer therapeutics [27-29], as altered and abnormal expression of HDACs has been discovered in different cancer types and linked to tumour development. HDAC inhibitors have low toxicity against normal cells and a capability to inhibit tumour cell growth at therapeutic concentrations, enabling them to be promising anticancer drugs in early phase clinical trials [30]. More details about these candidate compounds are described below to demonstrate their potential relevance in cancer, particularly in CRC treatment.

1. Trichostatin A is a classical HDAC inhibitor which has been revealed as a potent anti-cancer drug. A large amount of research has been carried out on trichostatin A and discovered that this compound inhibits the growth of bladder cancer, glioma, lung cancer, and pancreatic cancer cells [31-35]. It increases the sensitivity of chemotherapy drugs in gastric cancer cell lines [36]. In addition to inducing cell cycle arrest and apoptosis, this compound has function in inhibiting metastasis in colorectal cancer cells [37,38].

2. Vorinostat (suberoylanilide hydroxamic acid, SAHA) is classified as an HDAC inhibitor and used as an anticancer chemotherapy drug. It blocks the catalytic site of HDACs and has a significant anticancer activity against both hematologic and solid tumours. This compound causes growth arrest and induces apoptosis in a number of cancer cells, but with little or no toxic effects on normal cells [39]. It has undergone initial evaluation in Phase I clinical trial for patients with solid and hematologic malignancies [40,41], and was approved for treating cutaneous T-cell lymphoma [42].

3. Helminthosporium carbonum (HC)-toxin is an HDAC inhibitor in many organisms, including plants, insects, and mammals [43]. HC-toxin inhibits the malignant phenotype of primary neuroblastoma (NB) cells as well as established NB cell lines. It causes a shift to a benign and differentiated phenotype of NB cells via activating the retinoblastoma (RB) tumour suppressor network[44].

4. Sodium phenylbutyrate is an aromatic fatty acid known to be B-oxidized in vivo to phenylacetate, and is used to treat urea cycle disorders [45,46]. Sodium phenylbutyrate is an HDAC inhibitor under investigation as an anti-cancer agent, and in clinical trials of hemoglobinopathies, motor neuron diseases, and cystic fibrosis [46].

5. Mycophenolic acid (MPA) is an immunosuppressant drug used for the prevention of acute graft rejection in organ transplantation [47]. Through diverse molecular pathways and biological processes, MPA has an active role in anticancer activities [48]. This drug inhibits inosine monophosphate dehydrogenase (IMPDH) up-regulated in many tumours, and consequently inhibits proliferation, fibroblast and endothelial cell migration and induces apoptosis in cancer cells [49].

6. Irinotecan (Camptosar, Pfizer) is mainly used in colon cancer, particularly in combination with other chemotherapy agents. Irinotecan is the second line chemotherapy for advanced stage colorectal cancer. After surgical resection and adjuvant chemotherapy, the combination of oxaliplatin and 5-fluorouracil (5-FU) is used as first line treatment if colorectal cancer recurrence or metastasis occurred. Irinotecan is commenced if first line treatment failed and cancer progresses [50,51].

7. Etoposide (etoposide phosphate) is the first agent recognised as a topoisomerase II inhibiting anticancer drug. It derived from podophyllum plant which was found having antitumour activity against leukaemia. Approved by FDA because of its confirmed potent antineoplastic activity [52], etoposide is a widely used agent to treat various cancers including lung cancer, leukemias, non-Hodgking's lymphoma, testicular cancer, Kaposi's sarcoma, soft tissue sarcomas, and neuroblastoma. It is a standard therapy component to treat small cell lung cancer, testicular cancers and lymphomas [52,53]. The combination of etoposide and cisplatin is active in advanced CRCs and has been considered as a default option for treating patients with high-grade neuroendocrine tumours of the colon and rectum [53,54].

8. Valproic acid (VPA) is an HDAC inhibitor primarily used in the treatment of epilepsy and bipolar disorder [55]. It has been recognized as a promising anticancer drug with abundant literature support. For example, it was reported recently that the long term use of this compound is associated with reduced risk of head and neck cancer [56]. VPA has been suggested to play an important role in enhancing radiotherapy sensitivity of colon cancer cells, particularly with wildtype p53 genotype [57]. Through the down-regulation of the amyloid precursor protein, this compound inhibits proliferation of pancreatic and colon cancer cells [58].

9. Arachidonic acid (ARA) is a polyunsaturated fatty acid, whose metabolism plays important roles in cancer biology. The addition of exogenous ARA leads to growth inhibition and apoptosis of cancer cells including colon cancer cells [59,60]. Although the detailed mechanism has yet to be fully understood on how high levels of ARA induces apoptosis, it is clear that if the metabolic pathways that consume ARA (and hence lower its level) are coordinately activated, tumour growth is then promoted [59] through apoptotic escape [60]. These suggest that either ARA itself or specific inhibitors of ARA-metabolizing pathways may conform to some therapeutic benefit.

10. Rifabutin is a bactericidal antibiotic drug primarily used to treat tuberculosis as first-line treatment. It is among the list of anti-tuberculosis medicines recommended by the World Health Organization (the 18th WHO Model List of Essential Medicines April 2013) for use in patients with HIV receiving protease inhibitors [61]. Rifabutin has shown high efficacy in treating Helicobacter pylori infection, which is associated with a range of upper gastrointestinal diseases including gastric cancer [62]. Rifabutin has also been studied for treating Crohn's Disease, a type of incurable inflammatory bowel disease that may affect any part of the gastrointestinal tract including colon and rectum [63,64].

## Conclusions

The methods and procedure we have developed in this paper can serve as a paradigm for successful applications of connectivity mapping to identify promising candidate therapeutics in human diseases, and in particular cancers. On the one hand, the promising results we obtained suggest that genes on the signature list are highly relevant to colorectal cancer and they might be used as therapeutic targets in developing new treatments for the disease. On the other hand, the concept of connectivity mapping suggests that the drugs on the result list are likely to share some common molecular mechanisms in their actions against the disease. The fact that irinotecan and etoposide are currently used chemotherapeutic agents in colon cancer indicates that other drugs may also have similar activities or potentials.

In this work, we have used the CMap02 reference profiles as the core database to demonstrate the application of our methods in colorectal cancer. Recently, the LINCS project at the Broad Institute has massively expanded the compound collection to over 20,000, tested on 15 types of cells, generating over 1 million perturbation profiles. In principal, the methods developed here can be similarly applied using the expanded LINCS data as the reference database, which is likely to return additional drug connections to the CRC gene signature. With ongoing research in systems biology and bioinformatics addressing challenges presented by big data, it will become feasible to fully utilize the LINCS data and integrate them into the sscMap connectivity mapping framework.

In conclusion, the encouraging results obtained in the present study indicate that the multi-dataset method we proposed is an effective and robust way to generate high quality gene signatures for connectivity mapping, and the publication of the gene signature and drug hits obtained here will be beneficial to systems biology, cancer bioinformatics research communities and beyond.

## Competing interests

The authors declare that they have no competing interests.

## Authors' contributions

QW, PH and SDZ designed the study. QW collected the data, developed algorithms, and drafted the manuscript. QW, POR and PDD analysed the data. QW, ML, SVS, and MST interpreted the results. All authors contributed to writing the manuscript and provided technical input. All authors read and approved the final manuscript.

## Declarations

The funding for the publication of this article has been provided by BBSRC and Queen's University Belfast for open access.

This article has been published as part of *BMC Systems Biology *Volume 9 Supplement 5, 2015: Selected articles from the IEE International Conference on Bioinformatics and Biomedicine (BIBM 2014): Bioinformatics. The full contents of the supplement are available online at http://www.biomedcentral.com/bmcsystbiol/supplements/9/S5.

## References

[B1] Paul SM Mytelka DS Dunwiddie CT Persinger CC Munos BH Lindborg SR Schacht AL How to improve R&D productivity: the pharmaceutical industry's grand challenge Nature reviews Drug discovery 2010 9 3 203 214 20168317 10.1038/nrd3078

[B2] DiMasi JA Hansen RW Grabowski HG The price of innovation: new estimates of drug development costs Journal of health economics 2003 22 2 151-185 12606142 10.1016/S0167-6296(02)00126-1

[B3] Chong CR Jr DJS New uses for old drugs Nature 2007 448 7154 645-646 17687303 10.1038/448645a

[B4] Trevino V Falciani F Barrera-Saldana HA DNA microarrays: a powerful genomic tool for biomedical and clinical research Molecular medicine (Cambridge Mass.) 2007 13 9-10 527 541 10.2119/2006-00107.Trevino PMC1933257 17660860

[B5] Lamb J Crawford ED Peck D Modell JW Blat IC Wrobel MJ Lerner J Brunet JP Subramanian A Ross KN Reich M Hieronymus H Wei G Armstrong SA Haggarty SJ Clemons PA Wei R Carr SA Lander ES Golub TR The connectivity map: using gene-expression signatures to connect small molecules, genes, and disease Science (New York, N.Y.) 2006 313 5795 1929 1935 10.1126/science.1132939 17008526

[B6] Lamb J The connectivity map: a new tool for biomedical research Nature reviews Cancer 2007 7 1 54 60 10.1038/nrc2044 17186018

[B7] Qu XA Rajpal DK Applications of connectivity map in drug discovery and development Drug discovery today 2012 17 23-24 1289 1298 10.1016/j.drudis.2012.07.017 22889966

[B8] McArt DG Dunne PD Blayney JK Salto-Tellez M Schaeybroeck SV Hamilton PW Zhang SD Connectivity mapping for candidate therapeutics identification using next generation sequencing rna-seq data PloS one 2013 8 6 66902 10.1371/journal.pone.0066902 PMC3694114 23840550

[B9] Zhang SD Gant TW A simple and robust method for connecting small-molecule drugs using gene-expression signatures BMC bioinformatics 2008 9 258-21059258 18518950 10.1186/1471-2105-9-258 PMC2464610

[B10] Zhang SD Gant TW sscmap: an extensible java application for connecting small-molecule drugs using gene-expression signatures BMC bioinformatics 2009 10 236-210510236 19646231 10.1186/1471-2105-10-236 PMC2732627

[B11] McArt DG Bankhead P Dunne PD Salto-Tellez M Hamilton P Zhang SD cudaMap: a GPU accelerated program for gene expression connectivity mapping BMC Bioinformatics 2013 14 305 10.1186/1471-2105-14-305 24112435 PMC3852931

[B12] Chan SK Griffith OL Tai IT Jones SJ Meta-analysis of colorectal cancer gene expression profiling studies identifies consistently reported candidate biomarkers. Cancer Epidemiol Biomarkers Prev 2008 17 3 543 552 10.1158/1055-9965.EPI-07-2615 18349271

[B13] Siegel R Naishadham D Jemal A Cancer statistics, 2013 CA: a cancer journal for clinicians 2013 63 1 11 30 10.3322/caac.21166 23335087

[B14] Tsukamoto S Ishikawa T Iida S Ishiguro M Mogushi K Mizushima H Uetake H Tanaka H Sugihara K Clinical significance of osteoprotegerin expression in human colorectal cancer Clinical cancer research 2011 17 8 2444 2450 10.1158/1078-0432.CCR-10-2884 21270110

[B15] Sheffer M Bacolod MD Zuk O Giardina SF Pincas H Barany F Paty PB Gerald WL Notterman DA Domany E Association of survival and disease progression with chromosomal instability: a genomic exploration of colorectal cancer Proceedings of the National Academy of Sciences of the United States of America 2009 106 17 7131 7136 10.1073/pnas.0902232106 19359472 PMC2678450

[B16] Del Rio M Mollevi C Vezzio-Vie N Bibeau F Ychou M Martineau P Specific extracellular matrix remodeling signature of colon hepatic metastases PLoS ONE 2013 8 9 74599 10.1371/journal.pone.0074599 PMC3762755 24023955

[B17] Zhang SD Gant TW A statistical framework for the design of microarray experiments and effective detection of differential gene expression Bioinformatics 2004 20 16 2821 2828 10.1093/bioinformatics/bth336 15180939

[B18] Zhang SD Towards accurate estimation of the proportion of true null hypotheses in multiple testing PLoS ONE 2011 6 4 18874 10.1371/journal.pone.0018874 PMC3081301 21526119

[B19] Gorbatenko A Olesen CW Boedtkjer E Pedersen SF Regulation and roles of bicarbonate transporters in cancer Frontiers in physiology 2014 5 130 24795638 10.3389/fphys.2014.00130 PMC3997025

[B20] Tsukahara H Sekine K Uchiyama M Miura M Nakazato M Date Y Tsunezawa W Kotsuji F Nishida K Hiraoka M Mayumi M Uroguanylin level in umbilical cord blood Pediatrics international : official journal of the Japan Pediatric Society 2001 43 3 267 269 10.1046/j.1442-200x.2001.01393.x 11380922

[B21] Li BQ Huang T Liu L Cai YD Chou KC Identification of colorectal cancer related genes with mrmr and shortest path in protein-protein interaction network PloS one 2012 7 4 33393 10.1371/journal.pone.0033393 PMC3319543 22496748

[B22] Liu D Overbey D Watkinson LD Daibes-Figueroa S Hoffman TJ Forte LR Volkert WA Giblin MF In vivo imaging of human colorectal cancer using radiolabeled analogs of the uroguanylin peptide hormone Anticancer Research 2009 29 10 3777 3783 19846908

[B23] Birkenkamp-Demtroder K Maghnouj A Mansilla F Thorsen K Andersen CL Oster B Hahn S Orntoft TF Repression of kiaa1199 attenuates wnt-signalling and decreases the proliferation of colon cancer cells British journal of cancer 2011 105 4 552 561 10.1038/bjc.2011.268 21772334 PMC3170968

[B24] Iljin K Kilpinen S Ivaska J Kallioniemi O Edwards, D.R. Meta-Analysis of Gene Expression Microarray Data: Degradome Genes in Healthy and Cancer Tissues The cancer degradome : proteases and cancer biology 2008 Springer, New York ; London

[B25] McArt DG Zhang SD Identification of candidate small-molecule therapeutics to cancer by gene-signature perturbation in connectivity mapping PloS one 2011 6 1 16382 10.1371/journal.pone.0016382 PMC3031567 21305029

[B26] Bardou P Mariette J Escudie F Djemiel C Klopp C jvenn: an interactive Venn diagram viewer BMC Bioinformatics 2014 15 293 10.1186/1471-2105-15-293 25176396 PMC4261873

[B27] Ropero S Esteller M The role of histone deacetylases (HDACs) in human cancer Molecular oncology 2007 1 1 19 25 10.1016/j.molonc.2007.01.001 19383284 PMC5543853

[B28] Bolden JE Peart MJ Johnstone RW Anticancer activities of histone deacetylase inhibitors Nature reviews Drug discovery 2006 5 9 769 784 10.1038/nrd2133 16955068

[B29] Barneda-Zahonero B Parra M Histone deacetylases and cancer Molecular oncology 2012 6 6 579 589 10.1016/j.molonc.2012.07.003 22963873 PMC5528343

[B30] Lindemann RK Gabrielli B Johnstone RW Histone-deacetylase inhibitors for the treatment of cancer Cell cycle (Georgetown, Tex.) 2004 3 6 779 788 15153801

[B31] Li GC Zhang X Pan TJ Chen Z Ye ZQ Histone deacetylase inhibitor trichostatin a inhibits the growth of bladder cancer cells through induction of p21WAF1 and G1 cell cycle arrest International journal of urology 2006 13 5 581 586 10.1111/j.1442-2042.2006.01344.x 16771729

[B32] Wetzel M Premkumar DR Arnold B Pollack IF Effect of trichostatin a, a histone deacetylase inhibitor, on glioma proliferation in vitro by inducing cell cycle arrest and apoptosis Journal of neurosurgery 2005 103 6 Suppl 549 556 16383255 10.3171/ped.2005.103.6.0549

[B33] Kim HR Kim EJ Yang SH Jeong ET Park C Lee JH Youn MJ So HS Park R Trichostatin a induces apoptosis in lung cancer cells via simultaneous activation of the death receptor-mediated and mitochondrial pathway? Experimental & molecular medicine 2006 38 6 616 624 10.1038/emm.2006.73 17202837

[B34] Piacentini P Donadelli M Costanzo C Moore PS Palmieri M Scarpa A Trichostatin a enhances the response of chemotherapeutic agents in inhibiting pancreatic cancer cell proliferation Virchows Archiv 2006 448 6 797 804 10.1007/s00428-006-0173-x 16568310

[B35] Bai J Demirjian A Sui J Marasco W Callery MP Histone deacetylase inhibitor trichostatin a and proteasome inhibitor ps-341 synergistically induce apoptosis in pancreatic cancer cells Biochemical and biophysical research communications 2006 348 4 1245 1253 10.1016/j.bbrc.2006.07.185 16904634

[B36] Zhang X Yashiro M Ren J Hirakawa K Histone deacetylase inhibitor, trichostatin a, increases the chemosensitivity of anticancer drugs in gastric cancer cell lines Oncology reports 2006 16 3 563 568 16865256

[B37] Liu Y He G Wang Y Guan X Pang X Zhang B Mcm-2 is a therapeutic target of trichostatin a in colon cancer cells Toxicology letters 2013 221 1 23 30 10.1016/j.toxlet.2013.05.643 23770000

[B38] Meng J Zhang HH Zhou CX Li C Zhang F Mei QB The histone deacetylase inhibitor trichostatin a induces cell cycle arrest and apoptosis in colorectal cancer cells via p53-dependent and -independent pathways Oncology reports 2012 28 1 384 388 22552631 10.3892/or.2012.1793

[B39] Marks PA Discovery and development of saha as an anticancer agent Oncogene 2007 26 9 1351 1356 10.1038/sj.onc.1210204 17322921

[B40] Kelly WK O'Connor OA Krug LM Chiao JH Heaney M Curley T MacGregore-Cortelli B Tong W Secrist JP Schwartz L Richardson S Chu E Olgac S Marks PA Scher H Richon VM Phase I study of an oral histone deacetylase inhibitor, suberoylanilide hydroxamic acid, in patients with advanced cancer Journal of clinical oncology 2005 23 17 3923 3931 10.1200/JCO.2005.14.167 15897550 PMC1855284

[B41] Richon VM Garcia-Vargas J Hardwick JS Development of vorinostat: current applications and future perspectives for cancer therapy Cancer letters 2009 280 2 201 210 10.1016/j.canlet.2009.01.002 19181442

[B42] Marks PA Breslow R Dimethyl sulfoxide to vorinostat: development of this histone deacetylase inhibitor as an anticancer drug Nature biotechnology 2007 25 1 84 90 10.1038/nbt1272 17211407

[B43] Walton JD HC-toxin Phytochemistry 2006 67 14 1406 1413 10.1016/j.phytochem.2006.05.033 16839576

[B44] Deubzer HE Ehemann V Westermann F Heinrich R Mechtersheimer G Kulozik AE Schwab M Witt O Histone deacetylase inhibitor helminthosporium carbonum (HC)-toxin suppresses the malignant phenotype of neuroblastoma cells International journal of cancer 2008 122 8 1891 1900 10.1002/ijc.23295 18074352

[B45] Brusilow SW Phenylacetylglutamine may replace urea as a vehicle for waste nitrogen excretion Pediatric research 1991 29 2 147 150 10.1203/00006450-199102000-00009 2014149

[B46] Iannitti T Palmieri B Clinical and experimental applications of sodium phenylbutyrate Drugs in R&D 2011 11 3 227 249 10.2165/11591280-000000000-00000 21902286 PMC3586072

[B47] Millan O Oppenheimer F Brunet M Vilardell J Rojo I Vives J Martorell J Assessment of mycophenolic acid-induced immunosuppression: a new approach Clinical chemistry 2000 46 9 1376 1383 10973868

[B48] Dun B Sharma A Xu H Liu H Bai S Zeng L She JX Transcriptomic changes induced by mycophenolic acid in gastric cancer cells American journal of translational research 2013 6 1 28 42 24349619 PMC3853422

[B49] Dun B Sharma A Teng Y Liu H Purohit S Xu H Zeng L She JX Mycophenolic acid inhibits migration and invasion of gastric cancer cells via multiple molecular pathways PloS one 2013 8 11 81702 10.1371/journal.pone.0081702 PMC3829969 24260584

[B50] Weekes J Lam AK Sebesan S Ho YH Irinotecan therapy and molecular targets in colorectal cancer: a systemic review World journal of gastroenterology 2009 15 29 3597 3602 10.3748/wjg.15.3597 19653336 PMC2721232

[B51] Glimelius B Benefit-risk assessment of irinotecan in advanced colorectal cancer Drug safety 2005 28 5 417 433 10.2165/00002018-200528050-00005 15853443

[B52] Hande KR Etoposide: four decades of development of a topoisomerase II inhibitor European journal of cancer 1998 34 10 1514 1521 10.1016/S0959-8049(98)00228-7 9893622

[B53] Patta A Fakih M First-line cisplatin plus etoposide in high-grade metastatic neuroendocrine tumors of colon and rectum (MCRC NET): review of 8 cases Anticancer Research 2011 31 3 975 978 21498724

[B54] Passalacqua R Bisagni G Cocconi G Boni C Blasio BD Ceci G Cisplatin and etoposide in advanced colorectal carcinoma Annals of Oncology 1991 2 9 687 688 1742225 10.1093/oxfordjournals.annonc.a058050

[B55] Mologni L Cleris L Magistroni V Piazza R Boschelli F Formelli F Gambacorti-Passerini C Valproic acid enhances bosutinib cytotoxicity in colon cancer cells Int J Cancer 2009 124 8 1990 1996 10.1002/ijc.24158 19123474

[B56] Kang H Gillespie TW Goodman M Brodie SA Brandes M Ribeiro M Ramalingam SS Shin DM Khuri FR Brandes JC Long-term use of valproic acid in US veterans is associated with a reduced risk of smoking-related cases of head and neck cancer Cancer 2014 120 9 1394 1400 10.1002/cncr.28479 24664792 PMC4102261

[B57] Chen X Wong P Radany E Wong JY HDAC inhibitor, valproic acid, induces p53-dependent radiosensitization of colon cancer cells Cancer Biother Radiopharm 2009 24 6 689 699 10.1089/cbr.2009.0629 20025549 PMC2920759

[B58] Venkataramani V Rossner C Iffland L Schweyer S Tamboli IY Walter J Wirths O Bayer TA Histone deacetylase inhibitor valproic acid inhibits cancer cell proliferation via down-regulation of the alzheimer amyloid precursor protein J Biol Chem 2010 285 14 10678 10689 10.1074/jbc.M109.057836 20145244 PMC2856276

[B59] Cao Y Pearman AT Zimmerman GA McIntyre TM Prescott SM Intracellular unesterified arachidonic acid signals apoptosis Proc Natl Acad Sci USA 2000 97 21 11280 11285 10.1073/pnas.200367597 11005842 PMC17191

[B60] Monjazeb AM High KP Connoy A Hart LS Koumenis C Chilton FH Arachidonic acid-induced gene expression in colon cancer cells Carcinogenesis 2006 27 10 1950 1960 10.1093/carcin/bgl023 16704987

[B61] WHO: WHO model lists of essential medicines Technical report 2013 http://www.who.int/medicines/publications/essentialmedicines/en/index.html Accessed 28 May 2015

[B62] Gisbert JP Calvet X Review article: rifabutin in the treatment of refractory helicobacter pylori infection Alimentary Pharmacology & Therapeutics 2012 35 2 209 221 10.1111/j.1365-2036.2011.04937.x 22129228

[B63] Selby W Pavli P Crotty B Florin T Radford-Smith G Gibson P Mitchell B Connell W Read R Merrett M Ee H Hetzel D Two-year combination antibiotic therapy with clarithromycin, rifabutin, and clofazimine for Crohn's disease Gastroenterology 2007 132 7 2313 2319 10.1053/j.gastro.2007.03.031 17570206

[B64] Chamberlin W Importance of the Australian Crohn's disease antibiotic study Gastroenterology 2007 133 5 1744 1745 10.1053/j.gastro.2007.09.013 17983827

